# Five recurrent BRCA1/2 mutations are responsible for cancer predisposition in the majority of Slovenian breast cancer families

**DOI:** 10.1186/1471-2350-9-83

**Published:** 2008-09-10

**Authors:** Mateja Krajc, Erik Teugels, Janez Zgajnar, Guido Goelen, Nikola Besic, Srdjan Novakovic, Marko Hocevar, Jacques De Grève

**Affiliations:** 1 Institute of Oncology Ljubljana, Zaloska 2, 1000 Ljubljana, Slovenia; 2 Laboratory of Molecular Oncology, Department of Medical Oncology, Oncologisch Centrum UZ Brussel, Laarbeeklaan 101, 1090 Brussels, Belgium

## Abstract

**Background:**

Both recurrent and population specific mutations have been found in different areas of the world and more specifically in ethnically defined or isolated populations. The population of Slovenia has over several centuries undergone limited mixing with surrounding populations.

The current study was aimed at establishing the mutation spectrum of *BRCA1/2 *in the Slovenian breast/ovarian cancer families taking advantage of a complete cancer registration database. A second objective was to determine the cancer phenotype of these families.

**Methods:**

The original population database was composed of cancer patients from the Institute of Oncology Ljubljana in Slovenia which also includes current follow-up status on these patients. The inclusion criteria for the *BRCA1/2 *screening were: (i) probands with at least two first degree relatives with breast and ovarian cancer; (ii) probands with only two first degree relatives of breast cancer where one must be diagnosed less than 50 years of age; and (iii) individual patients with breast and ovarian cancer, bilateral breast cancer, breast cancer diagnosed before the age of 40 and male breast cancer without any other cancer in the family.

**Results:**

Probands from 150 different families met the inclusion criteria for mutation analysis of which 145 consented to testing. A *BRCA1/2 *mutation was found in 56 (39%). Two novel large deletions covering consecutive exons of *BRCA1 *were found. Five highly recurrent specific mutations were identified (1806C>T, 300T>G, 300T>A, 5382insC in the *BRCA1 *gene and IVS16-2A>G in the *BRCA2 *gene). The IVS16-2A>G in the *BRCA2 *gene appears to be a unique founder mutation in the Slovenian population. A practical implication is that only 4 PCR fragments can be used in a first screen and reveal the cancer predisposing mutation in 67% of the *BRCA1/2 *positive families. We also observed an exceptionally high frequency of 4 different pathogenic missense mutations, all affecting one of the cryptic cysteine residues of the *BRCA1 *Ring Finger domain.

**Conclusion:**

A high mutation detection rate and the frequent occurrence of a limited array of recurring mutations facilitate *BRCA1/2 *mutation screening in Slovenian families.

## Background

Breast cancer is the most common malignancy among women in developed countries. A family history of breast and/or ovarian cancer is the most important risk factor for the development of these cancers [1]. It is estimated that about 5 – 10% of breast cancer cases may be due to inherited predisposition [2]. In the context of high risk families studies have provided the evidence for at least two major cancer susceptibility genes: *BRCA1 *(17q21) [3] and *BRCA2 *(13q12) [4]. The cumulative risk for breast cancer for a woman carrying a *BRCA1 *or *BRCA2 *mutation is estimated to be as high as 85% by the age of 70 years and female carriers are also at a substantially increased risk of developing ovarian cancer [5].

Since the initial identification of both genes more than 3700 cancer predisposing mutations have been reported to the Breast Cancer Information Core (BIC) [6]. So far, most mutations have been identified in a single or a few families at most. However, several founder mutations are described in defined populations, such as the Ashkenazi Jewish [7], the Icelandic [8] and the Dutch population [9]. We have previously reported a highly recurrent *BRCA2 *founder mutation for the Slovene population [10]. Slovenia has two million inhabitants and every year close to 1100 women are diagnosed with breast cancer [11].

Genetic counselling for inherited breast/ovarian cancer in Slovenia started at the Institute of Oncology Ljubljana, Slovenia in October 1999. Until then the interest of Slovenian population (and health care workers) in cancer genetic counselling was unknown. The first step in implementing a *BRCA1/2 *genetic counselling program that includes mutation screening and then carrier detection in families found mutation-positive, was the approval of the Commission for Medical Ethics at the Ministry of Health, Republic of Slovenia.

The aim of our study was to assess: (i) the nature of *BRCA1/2 *mutations found in Slovenian population and (ii) the cancer phenotype in *BRCA1/2 *mutation positive families.

For anticipated cost-effectiveness issues, a mutation screen was only initiated in families that met minimal inclusion criteria with regard to cancer phenotype as detailed below [12].

## Methods

### Patients and families

This study was performed with families residing in Slovenia, and more specifically those followed at the Institute of Oncology of Ljubljana; the major centralized cancer centre in the country responsible for the national cancer registry. The accrual period was from October 1999 through March 2006. Cancer patients who were under surveillance at the Institute received a document with written information on familial cancer and a form in which they could detail the cancer diagnoses in the family. The questionnaire asked for detailed information in order to gather the required information about the cancer diagnoses and age of cancer incidence. This information was in addition cross – checked in the comprehensive database of the population cancer registry of the Institute, where accurate and detailed data on cancer types and age of cancer diagnoses were available. The cancer registry has a system of compulsory cancer registration since 1950.

Based on that information, probands were selected for screening according to the liberal inclusion criteria adapted from Brussels [12]: (i) probands with at least two first degree relatives with breast and ovarian cancer; (ii) probands with only two first degree relatives of breast cancer where one must be diagnosed less than 50 years of age; and (iii) individual patients with breast and ovarian cancer, bilateral breast cancer, breast cancer diagnosed before the age of 40 and male breast cancer without any other cancer in the family.

The multidisciplinary team supervising the process of counselling, screening and testing was composed of a surgical oncologist, a medical oncologist, a molecular biologist, a radiation oncologist, a radiologist, a gynaecologist, a geneticist, a cancer genetic counsellor and a research nurse.

After a mutation was found in the proband, all possible carriers in the family were offered, via the probands, genetic counselling and genetic testing, each time covered by an informed consent.

### Mutation screening

The mutation screen was performed in the Laboratory of Molecular Oncology at the Vrije Universiteit Brussel, on leucocyte DNA obtained from blood samples using the QIAamp DNA blood midi kit (Qiagen, Hilden, Germany). The full open reading frame of both *BRCA1 *and *BRCA2 *genes was analyzed in families with more than one ovarian or breast cancer case, except in families with only 2 breast cancer cases. In these families we restricted the mutation screen for cost-effectiveness reasons to the large exons of *BRCA1/2 *(by PTT) and the highly recurrent mutations identified in the course of the study (*BRCA1 *exons 5 and 20, *BRCA2 *exon 17). A restricted mutation analysis was also performed on isolated cases with bilateral breast cancer, isolated male breast cancer cases and isolated cases having developed breast and ovarian cancer.

The large exons (exon 11 from *BRCA1*, exons 10 and 11 from *BRCA2*) were analyzed by the Protein Truncation Test [13]. All small coding exons as well as the ends of the large exons were analyzed by Denaturing Gradient Gel Electrophoresis (Ingeny International, Goes, The Netherlands) [14]. A multiplex Ligand probe assay (MLPA) for the *BRCA1 *gene was performed with probe set P002 and confirmed with probe set P087 (MRC-Holland, Amsterdam, The Netherlands), each time on a batch of 8 samples. Data analysis (normalization and equalization) was performed using a self designed Excel spreadsheet. Samples revealing abnormal migration profiles, suggestive for the presence of a mutation were subjected to nucleotide sequencing (Sequenase Version 2.0 DNA sequencing kit from USB).

### Statistical analysis

Descriptive statistics were used for analysis of the data. Mean values and test for equality of means were calculated with the SPSS statistical software program.

## Results

**Figure 1:**
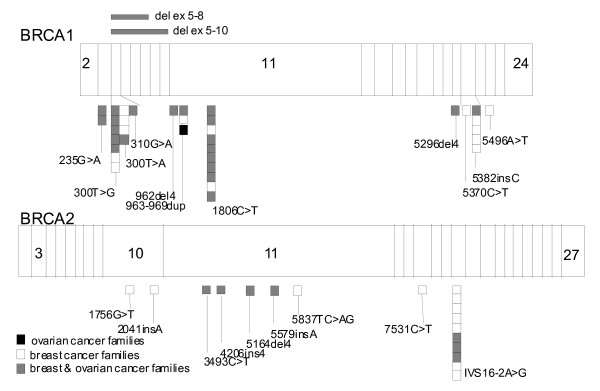
Identified *BRCA1/2 *mutations in Slovenian breast and/or ovarian cancer families.

Four hundred thirty seven cancer patients received the initial inquiries and 289 returned them of which finally probands from 150 (39%) different families matched the inclusion criteria. Only 5 (3.3%) of these declined further testing. A *BRCA1/2 *mutation screen was performed on 145 families and a cancer predisposing mutation was found in 56 (39%). The mutations were distributed over the whole length of the *BRCA1 *and *BRCA2 *coding sequences (Figure 1). After the cancer predisposing mutation was identified in the family, 95 additional family members from 36 of these families decided to have a test of which 40 were identified as carriers.

### BRCA1 mutation analysis

Thirteen distinct *BRCA1 *mutations were found in 38 families (Figure 1). Seven of the mutations were protein truncating, 4 missense mutations and 2 genomic rearrangements. The 4 different pathogenic missense found in fourteen families were located in exon 3 (235G>A) and exon 5 (300T>G, 300T>A, 310G>A). These mutations all affect one of the 7 RING domain cysteines that are crucial for correct binding of the Zn atoms. Several mutations were found repeatedly in different families. These include already reported 1806C>T *BRCA1 *mutation [6] found in 10 families; and the 5382insC *BRCA1 *mutation found in five families, which is the second most common reported mutation worldwide. The 967ins7 *BRCA1 *mutation was found three times. All other mutations, including 2 novel large deletions in *BRCA1*, one involving exons 5 to 8, the other exons 5 to 10, were detected only once (Figure 1).

### BRCA2 mutation analysis

Nine distinct *BRCA2 *mutations were identified in 18 families (Figure 1). Eight are protein truncating mutations, while one was a splice site mutation, the IVS16-2A>G. This mutation is often found in Slovenian breast cancer families (10 families) and was already reported by our group [10]. All other mutations found in BRCA2 gene were detected only once (Figure 1).

### Mutation detection rate

Although more families need to be investigated to reach statistical significance, the probability of finding a mutation correlated numerically with the number of affected patients in breast cancer only families: with 3 or less affected members mutations were found in 16/64 families (25%) compared to 10/21 (48%) when there were more than 3 affected family members.

However in families with ovarian cancer a mutation was found in 24/46 (52%) of families with less than four affected members and in 7/14 (50%) families with greater than three affected family members, indicating that the presence of ovarian cancer seems much stronger predictor for finding a *BRCA1/2 *mutation than the number of breast cancer cases in the family. The presence of recurrent mutations in this population permits the identification of cancer predisposing mutations in 67% of the *BRCA1/2 *mutant families by just analyzing 4 PCR fragments by DGGE. A screen restricted to these 4 fragments could therefore be performed on patients with a low probability for finding a *BRCA1/2 *mutation.

### Cancer phenotypes in families with a BRCA1 or BRCA2 mutation and genotypic-phenotypic correlations

The sample size and mixed inclusion criteria does not permit an accurate assessment of genotype-phenotype correlations, however some trends could be observed.

The ages at diagnosis as well as the number and types of cancer in each family with a mutation are shown in the additional file 1. The mean age at breast cancer diagnosis in *BRCA1 *mutation carriers was 42.98 years and 48.71 years for *BRCA2 *mutation carriers, respectively. By using T test for equality of means, difference between mean age in both groups at breast cancer diagnosis was statistically significant (p = 0.038).

The most frequent cancers in *BRCA1/2 *positive families are summarized in the table 1.

**Table 1:** Overall frequency of cancer types in 56 *BRCA1/2 *positive families

**No. of affected family members**	**BC**	**bil BC**	**MBC**	**OC**	**CC**	**GC**	**leukemia**	**pro Ca**	**pan Ca**	**Ca uterus**	**sarcoma**
**38 ***BRCA1 *positive families	62	17	0	33	14	4	5	2	4	6	2
**18 ***BRCA2 *positive families	59	7	4	7	5	3	3	1	4	0	0

OC – ovarian cancer, BC – breast cancer (female), bil – bilateral, MBC – male breast cancer, CC – colon cancer, GC – gastric cancer, pan – pancreas, pro – prostate, Ca – cancer, Nr. – number

Ovarian cancers (OC) were more often found in *BRCA1 *families compared with *BRCA2 *families. Consequently, a breast cancer only phenotype was found in a minority of *BRCA1 *mutation families (14 out of 38), but a majority of *BRCA2 *families (11 out of 18).

It is noteworthy that in our series the average number of breast cancers (BC) per family was twice as much in *BRCA2 *families (3.3) as compared to *BRCA1 *families (1.6).

Because of the occurrence of a few highly recurrent mutations, we had had the opportunity to estimate the relative risk for breast versus ovarian cancer in these particular families. In the 10 *BRCA1 *families with the 1806C>T mutation we counted 23 cases of BC compared to 11 cases of OC (relative risk: 2.1). For the 14 families with a missense mutation in the Ring-Finger domain the relative risk was quite similar (3.4; 41 BC and 12 OC cases). In the 10 families with the *BRCA2 *splice site mutation IVS16-2A>G the ratio of BC vs. OC was very high (49 BC and 3 OC; relative risk: 16.33). In our previous report we reported this as a BC only mutation. With the expansion of families a few OC were identified.

To get an estimate of the relative cancer penetrance among the three types of families with a recurrent cancer predisposing mutations we determined how many female first degree relatives of the index case above 18 years developed breast cancer (see additional file 1). This breast cancer incidence is 17.8% (13/73) and 15.1% (8/53) in *BRCA1 *families with a Ring domain missense mutation and 1806C>T, respectively. In *BRCA2 *IVS16-2A>G families we found a breast cancer incidence of 24.6% (15/61), which is higher than what is seen in the two types of *BRCA1 *families. The penetrance of ovarian cancer is much lower in IVS16-2A>G *BRCA2 *families than in *BRCA1 *families with a Ring domain missense mutation or the 1806C>T mutation.

## Discussion

Slovenia is a central European area country and despite a complex history of occupation throughout history and the fact that it has been an independent country for only 17 years, the population has preserved its own language and culture for centuries. Currently 83% of the population is considered of Slovene origin. Most Slovenes live in the current Slovenia, but due to variations in the size of the country significant Slovene populations also live in surrounding countries especially Austria, northern Italy and also some Balkan countries.

In this report we tested 145 breast and/or ovarian cancer families in Slovenia for *BRCA1/2 *gene mutations.

There was a striking high interest for genetic testing after providing systematic and standardized information using written material. Only 5 probands (3.3%) from 150 families that met our inclusion criteria for screening refused mutation analysis. This level of participation is considerably higher than some report in the literature, where around 50% of eligible probands opt for screening [15]. It seems that the information about the screening was communicated on the adequate level of understanding. The high participation rate of probands contrasted sharply with the subsequent low interest in counselling of family members. Only an average of two possible or probable carriers per *BRCA 1/2 *mutation positive family came forward spontaneously to request testing. The reasons for this lack of interest are not known, as we did not investigate this on our population. Adequate proband-based information dissemination in a proband mediated model was identified in Belgium by Sermijn et al. as highly anomalous [16]. That study showed that in reality the interest in counselling and testing was high as almost all relatives wanted to be further informed about the various aspects concerning hereditary breast/ovarian cancer and also subsequently requested a genetic test after being properly informed. This also agrees with our high participation rate observed in the properly informed probands.

Since we often face genetic counselling for members of small families, we applied minimal selection criteria before initiating a search for *BRCA1*/*2 *mutations. Despite these liberal criteria the overall mutation detection rate (MDR) was 39% (56/145 screened families), which is high when compared to what was previously reported for other populations where the MDR are between 15% and 37% with usually more stringent selection criteria with regard to familial cancer phenotype than in our study [17-21]. The intake criteria we employed thus seemed adequate and practical for further use in our population [12]. It is also possible that with our mutation screening methodology we did not detect all possible mutations and thus we have a lower estimate of the mutation rate in our population. For the same reason the actual mutation heterogeneity could be greater than we estimate.

It is known and was also observed in the current series, that the best MDR were obtained in families with either a high number of breast cancer cases or families that also include ovarian cancers, in which case the number of breast cancers is of lesser importance. Such a high MDR of > 50% in families that also include at least one ovarian cancer case has also been observed in other populations [12]. However, more Slovene breast cancer only families were found mutation-positive as compared with comparable Belgian families. Perhaps the high MDR in this type of families can be explained by the occurrence of a highly recurrent founder mutation in the Slovene families that predispose almost exclusively and at high penetrance for breast cancer (IVS16-2A>G in *BRCA2*) since ignoring the families with this mutation leads to comparable MDR for both populations.

We found 2 novel large deletions involving exons 5–8 and 5–10 of *BRCA1 *that probably occurred through a recombination event between misaligned repetitive elements (Alu repeats) abundant within the intron sequences of *BRCA1*.

Four distinct types of mutations occurred very frequently in the Slovene population. In fact, the analysis of only 4 PCR fragments (1806C>T, exon 5 (300T>G, 300T>A), 5382insC in the *BRCA1 *gene and IVS16-2A>G in the *BRCA2 *gene) would lead to the identification of the cancer predisposing mutations in 67% of the *BRCA1/2 *mutation-positive families. Therefore the genetic screening was initiated with the detection of these four particular DNA regions. As a result, families that do not strictly meet the including criteria for the genetic screen can be submitted to a restricted analysis when cost-effectiveness is an issue and a mutation screen would otherwise not be engaged.

Earlier in the current project we discovered a Slovenian founder mutation in three of the first seven screened families (*BRCA2 *splice site mutation IVS16-2A>G) [10]. This mutation was found in the current series in 10/56 (18%) of all families carrying a *BRCA1/2 *mutation and is together with the world wide recurrent 1806C>T mutation in *BRCA1 *gene the most common mutation in Slovenian population. According to the BIC database IVS16-2A>G was reported worldwide only three times, twice by Myriad in "Western Europeans" and once by Santarosa from Aviano, an Italian city only 50 km from Slovenian border [22,23].

The *BRCA1 *mutation 1806C>T was found in 10/56 *BRCA 1/2 *positive families (18%). This nonsense mutation clusters mainly in Sweden, and is reported as a Swedish founder mutation. The mutation however has also been found in Belgian and Spanish families, and according to the BIC database in German, Austrian, Dutch, Danish and Italian ethnic populations as well [24-26].

We found an exceptionally high proportion (37%) of pathogenic missense mutations in the RING Finger domain. The RING motif is characterized by a conserved pattern of 7 cysteine and 1 histidine residues arranged in an interleaved fashion forming two distinct Zn^2+ ^binding sites termed Site I and Site II [27]. Interestingly, each of the 3 cysteines involved in Site II is affected by one of the 4 identified missense mutations. One of these mutations, 300T>A, was found in 5 families and has not been reported elsewhere. According to the data available in BIC database and from the literature, such a high frequency and clustering of RING Finger domain missense mutations is reported Italy, our neighbouring country [6] and in Czech Republic, Latvia, Poland, Hungary [28-31].

As the Slovene population mixed to some extent with the inhabitants of neighbouring countries (Austria, Italy, Hungary and Croatia) we can assume that we also share some *BRCA1/2 *mutations with these populations. Indeed, all three most common mutations found (except for the Slovenian founder mutation) in Slovenians segregate in Italian and Austrian families [6,32-34]. Unfortunately, there is a lack of data on *BRCA1/2 *mutations from the remainder of the Balkan region. There were only few reports of *BRCA1/2 *mutation screening in the region of former Yugoslavia [35] and by comparing results the only common mutation found was *BRCA1 *5382insC, which is known as an Ashkenazi Jewish mutation that occurs frequently in the Central and Eastern Europe (Czech Republic, Lithuania, Poland, Slovakia, Russia) [28,30]. This mutation accounts for 5/56 (9%) positive families in our series.

With regard to familial cancer phenotype this relatively small cohort does not allow for the uncovering of subtle differences between the *BRCA1*/*2 *mutant families, except for the double incidence rate of ovarian cancers in *BRCA1 *compared to *BRCA2 *positive families as reported widely before. Families with inherited mutations in the *BRCA2 *gene give rise to a multi-site cancer phenotype, which includes besides breast cancer (in females and males), ovarian, colon, stomach, pancreatic, prostate and laryngeal cancer, as reported before [36]. In our sample uterine cancer was related to the *BRCA1 *gene mutation. Both *BRCA1 *and *BRCA2 *mutation positive families included cases of leukemia that accounted for about 2 percent of affected individuals. In one family with 5382insC *BRCA1 *gene mutation, 2 cases with sarcoma were reported. Interpretation of these results and risk assessment for these other cancers is difficult in the view of the low penetrance for these other cancers.

It is known that the risk for ovarian cancer might vary depending on the location of the mutation within the *BRCA1/2 *coding sequence although this information is generally not used in the counselling process and does not appear in any national guidelines broadly used [37,38]. The high rate of specific recurrent mutations seen in the Slovene population allowed us to assess the relative risk for breast and ovarian cancer for these particular mutations [39]. The missense mutations in the RING domain and a protein truncating mutation in exon 11 (1806C>T), all located in *BRCA1*, induce comparable relative risks for breast versus ovarian cancer (3.4 and 2.1 respectively), while the *BRCA2 *IVS16-2G>A mutation appears to be highly predisposing for breast cancer compared to ovarian cancer. However, due to the small sample size these results should be confirmed in a larger sample set.

## Conclusion

In summary, *BRCA1/2 *mutation testing and counselling met with a high acceptance rate in Slovenia and with a high interest level in probands.

A high mutation detection rate and the frequent occurrence of a limited array of recurring mutations allow a simple and fast initial test for *BRCA1/2 *mutation screening in families with Slovenian ancestry.

## Competing interests

The authors declare that they have no competing interests.

## Authors' contributions

MK, JDG, ET, JZ, GG, NB, SN and MH designed the study, collected and analyzed the data and wrote the paper.

## Pre-publication history

The pre-publication history for this paper can be accessed here:



## Supplementary Material

Additional file 1 Cancer phenotypes of families with a *BRCA1/2 *mutation: *A. BRCA1 *mutation families phenotypes. *B. BRCA2 *mutation families phenotypes. Click here for file

## References

[B1] Lynch HT, Fain PR, Golgar D, Albano WA, Mailliard JA, McKenna P (1981). Familial breast cancer and its recognition in an oncology clinic. Cancer.

[B2] Ford D, Easton DF, Stratton M, Narod S, Goldgar D, Devilee P, Bishop DT, Weber B, Lenoir G, Chang-Claude J, Sobol H, Teare MD, Struewing J, Arason A, Scherneck S, Peto J, Rebbeck TR, Tonin P, Neuhausen S, Barkardottir R, Eyfjord J, Lynch H, Ponder BAJ, Gayther SA, Birch, Lindblom A, Stoppa-Lyonnet D, Bignon Y, Borg A, Hamann U, Haites N, Scott RJ, Maugard CM, Vasen H, Seitz S, Cannon-Albright LA, Schofield A, Zelada-Hedman M, the Breast Cancer Linkage Consortium (1998). Genetic heterogeneity and penetrance analysis of the BRCA1 and BRCA2 genes in breast cancer families. The Breast Cancer Linkage Consortium. Am J Hum Genet.

[B3] Miki Y, Swensen J, Shattuck-Eidens D, Futreal PA, Harshman K, Tavtigian S, Liu Q, Cochran C, Bennett LM, Ding W (1994). A strong candidate for the breast and ovarian cancer susceptibility gene BRCA1. Science.

[B4] Wooster R, Bignell G, Lancaster J, Swift S, Seal S, Mangion J, Collins N, Gregory S, Gumbs C, Micklem G (1995). Identification of the breast cancer susceptibility gene BRCA2. Nature.

[B5] Burke W, Daly M, Garber J, Botkin J, Kahn MJ, Lynch P, McTiernan A, Offit K, Perlman J, Petersen G, Thomson E, Varricchio C (1997). Recommendations for follow-up care of individuals with an inherited predisposition to cancer. II. BRCA1 and BRCA2. Cancer Genetics Studies Consortium. JAMA.

[B6] Breast Cancer Information Core. http://research.nhgri.nih.gov/bic/.

[B7] Struewing JP, Hartge P, Wacholder S, Baker SM, Berlin M, McAdams M, Timmerman MM, Brody LC, Tucker MA (1997). The risk of cancer associated with specific mutations of BRCA1 and BRCA2 among Ashkenazi Jews. N Engl J Med.

[B8] Thorlacius S, Olafsdottir G, Tryggvadottir L, Neuhausen S, Jonasson JG, Tavtigian SV, Tulinius H, Ogmundsdottir HM, Eyfjord JE (1996). A single BRCA2 mutation in male and female breast cancer families from Iceland with varied cancer phenotypes. Nat Genet.

[B9] Petrij-Bosch A, Peelen T, van Vliet M, van Eijk R, Olmer R, Drusedau M, Hogervorst FB, Hageman S, Arts PJ, Ligtenberg MJ, Meijers-Heijboer H, Klijn JG, Vasen HF, Cornelisse CJ, van't Veer LJ, Bakker E, van Ommen GJ, Devilee P (1997). BRCA1 genomic deletions are major founder mutations in Dutch breast cancer patients. Nat Genet.

[B10] Krajc M, De Greve J, Goelen G, Teugels E (2002). BRCA2 founder mutation in Slovenian breast cancer families. Eur J Hum Genet.

[B11] Cancer incidence in Slovenia 2004 (2007).

[B12] Goelen G, Teugels E, Bonduelle M, Neyns B, De Greve J (1999). High frequency of BRCA1/2 germline mutations in 42 Belgian families with a small number of symptomatic subjects. J Med Genet.

[B13] Hogervorst FB, Cornelis RS, Bout M, van VM, Oosterwijk JC, Olmer R, Bakker B, Klijn JG, Vasen HF, Meijers-Heijboer H (1995). Rapid detection of BRCA1 mutations by the protein truncation test. Nat Genet.

[B14] Hout AH Van der, Ouweland AM van den, Luijt RB van der, Gille HJ, Bodmer D, Bruggenwirth H, Mulder IM, Vlies P van der, Elfferich P, Huisman MT, ten Berge AM, Kromosoeto J, Jansen RP, van Zon PH, Vriesman T, Arts N, Lange MB, Oosterwijk JC, Meijers-Heijboer H, Ausems MG, Hoogerbrugge N, Verhoef S, Halley DJ, Vos YJ, Hogervorst F, Ligtenberg M, Hofstra RM (2006). A DGGE system for comprehensive mutation screening of BRCA1 and BRCA2: application in a Duch cancer clinic setting. Hum Mutat.

[B15] Armstrong K, Calzone K, Stopfer J, Fitzgerald G, Coyne J, Weber B (2000). Factors associated with decisions about clinical BRCA1/2 testing. Cancer Epidemiol Biomarkers Prev.

[B16] Sermijn E, Goelen G, Teugels E, Kaufman L, Bonduelle M, Neyns B, Poppe B, De Paepe A, De Greve J (2004). The impact of proband mediated information dissemination in families with a BRCA1/2 gene mutation. J Med Genet.

[B17] Yazici H, Bitisik O, Akisik E, Cabioglu N, Saip P, Muslumanoglu M, Glendon G, Bengisu E, Ozbilen S, Dincer M, Turkmen S, Andrulis IL, Dalay N, Ozcelik H (2000). BRCA1 and BRCA2 mutations in Turkish breast/ovarian families and young breast cancer patients. Br J Cancer.

[B18] Osorio A, Barroso A, Martinez B, Cebrian A, San Roman JM, Lobo F, Robledo M, Benitez J (2000). Molecular analysis of the BRCA1 and BRCA2 genes in 32 breast and/or ovarian cancer Spanish families. Br J Cancer.

[B19] Santarosa M, Viel A, Dolcetti R, Crivellari D, Magri MD, Pizzichetta MA, Tibiletti MG, Gallo A, Tumolo S, Del TL, Boiocchi M (1998). Low incidence of BRCA1 mutations among Italian families with breast and ovarian cancer. Int J Cancer.

[B20] Couch FJ, DeShano ML, Blackwood MA, Calzone K, Stopfer J, Campeau L, Ganguly A, Rebbeck T, Weber BL (1997). BRCA1 mutations in women attending clinics that evaluate the risk of breast cancer. N Engl J Med.

[B21] Hamann U, Haner M, Stosiek U, Bastert G, Scott RJ (1997). Low frequency of BRCA1 germline mutations in 45 German breast/ovarian cancer families. J Med Genet.

[B22] Santarosa M, Dolcetti R, Magri MD, Crivellari D, Tibiletti MG, Gallo A, Tumolo S, Della PL, Furlan D, Boiocchi M, Viel A (1999). BRCA1 and BRCA2 genes: role in hereditary breast and ovarian cancer in Italy. Int J Cancer.

[B23] Miolo G, Puppa LD, Santarosa M, De Giacomi C, Veronesi A, Bidoli E, Tibiletti MG, Viel A, Dolcetti R (2006). Phenotypic features and genetic characterization of male breast cancer families: identification of two recurrent BRCA2 mutations in north-east of Italy. BMC Cancer.

[B24] Johannsson OT, Staff S, Vallon-Christersson J, Kytola S, Gudjonsson T, Rennstam K, Hedenfalk IA, Adeyinka A, Kjellen E, Wennerberg J, Baldetorp B, Petersen OW, Olsson H, Oredsson S, Isola J, Borg A (2003). Characterization of a novel breast carcinoma xenograft and cell line derived from a BRCA1 germ-line mutation carrier. Lab Invest.

[B25] De Benedetti  V, Radice P, Pasini B, Stagi L, Pensotti V, Mondini P, Manoukian S, Conti A, Spatti G, Rilke F, Pierotti MA (1998). Characterization of ten novel and 13 recurring BRCA1 and BRCA2 germline mutations in Italian breast and/or ovarian carcinoma patients. Mutations in brief no. 178. Online. Hum Mutat.

[B26] Santarosa M, Viel A, Dolcetti R, Crivellari D, Magri MD, Pizzichetta MA, Tibiletti MG, Gallo A, Tumolo S, Del TL, Boiocchi M (1998). Low incidence of BRCA1 mutations among Italian families with breast and ovarian cancer. Int J Cancer.

[B27] Borden KL, Freemont PS (1996). The RING finger domain: a recent example of a sequence-structure family. Curr Opin Struct Biol.

[B28] Pohlreich P, Zikan M, Stribrna J, Kleibl Z, Janatova M, Kotlas J, Zidovska J, Novotny J, Petruzelka L, Szabo C, Matous B (2005). High proportion of recurrent germline mutations in the BRCA1 gene in breast and ovarian cancer patients from the Prague area. Breast Cancer Res.

[B29] Tikhomirova L, Sinicka O, Smite D, Eglitis J, Hodgson SV, Stengrevics A (2005). High prevalence of two BRCA1 mutations, 4154delA and 5382insC, in Latvia. Fam Cancer.

[B30] Janiszewska H, Haus O, Lauda-Swieciak A, Pasińska M, Laskowski R, Szymański W, Górski B, Lubiński J (2003). Frequency of three BRCA1 gene founder mutations in breast/ovarian cancer families from the Pomerania-Kujawy region of Poland. Clin Genet.

[B31] Looij M Van Der, Szabo C, Besznyak I, Liszka G, Csokay B, Pulay T, Toth J, Devilee P, King MC, Olah E (2000). Prevalence of founder BRCA1 and BRCA2 mutations among breast and ovarian cancer patients in Hungary. Int J Cancer.

[B32] Baudi F, Quaresima B, Grandinetti C, Cuda G, Faniello C, Tassone P, Barbieri V, Bisegna R, Ricevuto E, Conforti S, Viel A, Marchetti P, Ficorella C, Radice P, Costanco F, Venuta S (2001). Evidence of a founder mutation of BRCA1 in a highly homogeneous population from southern Italy with breast/ovarian cancer. Hum Mutat.

[B33] Ottini L, D'Amico C, Noviello C, Lauro S, Lalle M, Fornarini G, Colantuoni OA, Pizzi C, Cortesi E, Carlini S, Guadagni F, Bianco AR, Frati L, Contegiacomo A, Mariani-Costantini R (2000). BRCA1 and BRCA2 mutations in central and southern Italian patients. Breast Cancer Res.

[B34] Turchetti D, Cortesi L, Federico M, Bertoni C, Mangone L, Ferrari S, Silingardi V (2000). BRCA1 mutations and clinicopathological features in a sample of Italian women with early-onset breast cancer. Eur J Cancer.

[B35] Papp J, Raicevic L, Milasin J, Dimitrijevic B, Radulovic S, Olah E (1999). Germline mutation analysis of BRCA1 and BRCA2 genes in Yugoslav breast/ovarian cancer families. Oncol Rep.

[B36] Lubinski J, Phelan CM, Ghadirian P, Lynch HT, Garber J, Weber B, Tung N, Horsman D, Isaacs C, Monteiro AN, Sun P, Narod SA (2004). Cancer variation associated with the position of the mutation in the BRCA2 gene. Fam Cancer.

[B37] Gayther SA, Warren W, Mazoyer S, Russell PA, Harrington PA, Chiano M, Seal S, Hamoudi R, van Rensburg EJ, Dunning AM, Love R, Evans G, Easton D, Clayton D, Stratton MR, Ponder BA (1995). Germline mutations of the BRCA1 gene in breast and ovarian cancer families provide evidence for a genotype-phenotype correlation. Nat Genet.

[B38] Gayther SA, Mangion J, Russell P, Seal S, Barfoot R, Ponder BA, Stratton MR, Easton D (1997). Variation of risks of breast and ovarian cancer associated with different germline mutations of the BRCA2 gene. Nat Genet.

[B39] Novakovic S, Stegel V (2005). Rapid detection of most frequent Slovenian germ-line mutations in *BRCA1 *gene using real-time PCR and melting curve analysis. Radiol Oncol.

