# Supplementary material for: Five recurrent BRCA1/2 mutations are responsible for cancer predisposition in the majority of Slovenian breast cancer families
Source: BMC Med Genet. 2008 Sep 10;9:83. doi: 10.1186/1471-2350-9-83 (PMC2547096; doi:10.1186/1471-2350-9-83)
Supplement: Additional file 1 — Cancer phenotypes of families with a BRCA1/2 mutation: A. BRCA1 mutation families phenotypes. B. BRCA2 mutation families phenotypes. [file 1471-2350-9-83-S1.doc]

ADDITIONAL FILE

Additional file 1 - Cancer phenotypes of families with a *BRCA1/2* mutation:

1. *BRCA1* mutation families phenotypes
2. *BRCA2* mutation families phenotypes

A. *BRCA1* mutation families phenotypes

| Family Nr. | Proband gender, diagnosis | Proband age at diagnosis | Inclusion criteria classification* | Mutation **found – *BRCA1*** | **No. of first degree relatives with any type of cancer cancer above 18 years/ No. of all first degree rel. above 18 yrs (probands not included)** | All affected relatives (age at diagnosis), first-degree relatives in **bold** (probands not included) |
| --- | --- | --- | --- | --- | --- | --- |
| 1 | F, OC | 54 | i | 1806C>T | 5/10 | 4BC (50,42, 69,-),1 OC (50), 1CC (56), tongue Ca(45) |
| 2 | F, BC | 25 | i | 1806C>T | 1/3 | 1 OC(47) 1pan Ca(61), 1CC(-), 1GC (60) |
| 3 | F, BC | 50 | i | 1806C>T | 2/6 | 2BC(36,49),1 liver Ca(-),1throatCa (-) |
| 4 | F, no cancer | - | i | 1806C>T | 1/3 | 1BC+OC(34,48), 1BC (34), 1 OC (62) |
| 5 | F, OC | 32 | i | 1806C>T | 0/3 | 1 OC (54), 1BC (45), 2LC (63,-), 1 throat Ca (53) |
| 6 | F, BC+OC | 53, 56 | iii | 1806C>T | 1/10 | 2CC (50,74) |
| 7 | F, BC+OC | 62,62 | i | 1806C>T | 1/4 | 1BC (53) |
| 8 | F, OC | 48 | i | 1806C>T | 2/6 | 4BC (38,48,53,61) |
| 9 | F, BC | 49 | i | 1806C>T | 1/5 | 1 cervical cancer (37), 1 bilBC(30, 33), 1Ca uteri (45) |
| 10 | F, BC | 40 | i | 1806C>T | 2/3 | 1 OC+BC (46, 55) |
| 11 | F, BC | 50 | i | 300T>G | 2/7 | 1 OC(52),2BC (64,70), 1Ca uteri (50) |
| 12 | F, OC+ bil BC | 75, 39,52 | i | 300T>G | 2/8 | 1OC (55), 1CC (-) |
| 13 | F, BC | 46 | i | 300T>G | 3/6 | 1bil BC (49,62), 1BC (79), 4CC (42,66,78,88), 2GC (96,-), 1 pan Ca (57), 1 pro Ca (70) |
| 14 | F, bil BC | 34,43 | i | 300T>G | 2/6 | 1 OC (36), 1BC (50), 1 pro Ca (63),1 esophageal Ca (52) |
| 15 | F, OC | 69 | i | 300T>G | 1/8 | 1BC (38) |
| 16 | F, BC | 52 | i | 300T>G | 2/6 | 1BC(46), 1OC (46), 1 OC+BC(70,72) |
| 17 | F, BC | 31 | i | 300T>G | 1/4 | 1 bil BC (40, 43), 1 BC (60) |
| 18 | F, no cancer | - | i | 300T>A | 1/5 | 1bil BC (40,40), 2BC (50,70) |
| 19 | F, BC | 55 | ii | 300T>A | 1/7 | 1BC (39) |
| 20 | F, bil BC | 36,40 | iii | 300T>A | (-) | Ca larynx (-) |
| 21 | F, bil BC+OC | 38, 48, 52 | i | 300T>A | 3/7 | 1 BC+OC (-,-), 1 BC (68), 1 liver Ca (80), 1GC(70) |
| 22 | F, BC | 45 | i | 310G>A | 1/2 | 1 0C (60), 1BC (40), 1CC (-), 2 leukemia (20,62) |
| 23 | F, BC | 23 | i | 5382insC | 3/9 | 2BC (34,-), 1CC (-), 1 leukemia (58), 1MM (25), 2 sarcoma (-,-) |
| 24 | F, bil BC | 33,46 | i | 5382insC | 2/7 | 1 OC (50), 1CC (45) |
| 25 | F, bil BC | 34,36 | i | 5382insC | 0/5 | 1BC (44), 1Ca uteri (71) |
| 26 | F, bil BC | 36, 40 | iii | 5382insC | 1/8 | 1 leukemia (46) |
| 27 | F, BC | 34 | iii | 5382insC | 0/3 | 2 CC (58, 58), 1 cervical cancer (49) |
| 28 | F, OC | 57 | i | 967ins7 | 1/5 | 1 OC (68), 1GC (73), 1bladder Ca (61), 1LC (-) |
| 29 | F, BC | 52 | i | 967ins7 | 4/9 | 2 BC (33,49), 1 BC+panCa (60,60) |
| 30 | F, bil BC | 41, 42 | iii | 967ins7 | 1/5 | 2 LC (63, 67), 2 OC (41, 50), 1panCa(69) |
| 31 | F, bil BC+OC | 32, 38, 62 | i | 235G>A | 1/- | 1 BC (45) |
| 32 | F, bil BC+OC | 48,50,67 | i | 235G>A | 2/7 | 2 BC (32, 45) |
| 33 | F, bil BC | 50,66 | i | 5296del4 | 2/9 | 2 BC (39,41), 1 OC (36) |
| 34 | F, bil BC | 40, 52 | iii | 5370C>T | 0/6 | no other Ca in the family |
| 35 | F, BC | 32 | i | 5496A>T | 1/2 | 2 bil BC (35+41, -), 1 leukemia (40) |
| 36 | F, BC | 35 | i | 962del4 | 1/3 | 1 OC+bil BC (61,63,66) |
| 37 | F, OC | 54 | i | Del ex 5-8 | 5/10 | 3 OC (37, 55, 61), 2 BC (38, 53) |
| 38 | F, BC | 47 | ii | Del ex5-10 | 1/5 | 1BC+Ca uteri (54,67), 1OC(22), 2 Ca uteri (46,-), 1BC(34) |

NOTE. F - female, M- male, No. – number, OC - ovarian cancer, BC - breast cancer (female), MM - malignant melanoma, CC - colon cancer, pan - pancreas, GC - gastric cancer, pro - prostate, bil – bilateral, LC - lung cancer, Ca - cancer, (-) no data available, (+) in the same individual

* Inclusion criteria classification:

1. probands from families with at least two first degree relatives with breast and ovarian cancer;
2. probands from families with only two first degree relatives of breast cancer where one must be diagnosed less than 50 years of age and;
3. individual patients with breast and ovarian cancer, bilateral breast cancer, breast cancer diagnosed before the age of 40 and male breast cancer without any other cancer in the family.

B. *BRCA2* mutation families phenotypes

| Family Nr. | Proband gender, diagnosis | Proband age at diagnosis | Inclusion criteria classification* | Mutation **found – *BRCA2*** | **No. of first degree relatives with any type of cancer cancer above 18 years/ No. of all first degree rel. above 18 yrs (probands not included)** | All affected relatives (age at diagnosis), first-degree relatives in **bold** (probands not included) |
| --- | --- | --- | --- | --- | --- | --- |
| 39 | F, BC | 30 | iii | IVS 16-2A>G | 1/3 | 2 MBC (64,65), 1 OC(-), 6BC (68,79,-,-,-,-) |
| 40 | F, BC | 29 | ii | IVS 16-2A>G | 0/4 | 1BC (42) |
| 41 | F, bil BC | 51,53 | i | IVS 16-2A>G | 3/6 | 3BC (36,60,64), 1 throat Ca (-), 1LC (50) |
| 42 | F, BC | 55 | i | IVS 16-2A>G | 3/7 | 7 BC (32,39,39,40,42,43,45), 1 bil BC (50,57), 2CC (50,74), 1 pan Ca (49) |
| 43 | F, BC | 38 | i | IVS 16-2A>G | 1/3 | 2 BC (41,45), 1 bil BC+pan Ca (48,52,66), 1 LC (70) |
| 44 | F, OC+ bil BC | 39,58,58 | i | IVS 16-2A>G | 1/8 | 2BC (58,68), pan Ca (60), 1 Ca thyroid (-) |
| 45 | F, BC | 60 | i | IVS 16-2A>G | 4/10 | 3BC (33,50,51), 1CC (62),1 leukemia (65) |
| 46 | F, bil BC | 55,60 | i | IVS 16-2A>G | 2/11 | 2BC (50,51), 1 pro Ca (50) |
| 47 | F, BC | 54 | i | IVS 16-2A>G | 1/4 | 1BC+OC (53,73), 3BC (54,42,-), 1CC (-), GC (85) |
| 48 | M, MBC | 56 | iii | IVS 16-2A>G | 2/5 | 2 papilar thyroid ca (25, 40) |
| 49 | F, BC | 51 | i | 1756G>T | 3/9 | 1 BC+GC (30, 68), 1 BC (38), 1 leukemia (67) |
| 50 | M, no Ca | - | i | 2041insA | 3/5 | 5BC (36,43,55,60,73), 1LC(60) |
| 51 | F, BC+OC | 35,50 | i | 3493C>T | 3/6 | 1BC (39), 1GC (44), 1 cervical cancer (22) |
| 52 | M, MBC | 50 | iii | 4206ins4 | 1/5 | 1 OC (40), 1BC (59) |
| 53 | F, BC | 33 | i | 5164del4 | 1/2 | 1 OC (65) |
| 54 | F,OC+BC | 73,74 | iii | 5579insA | 0/3 | no other Ca in the family |
| 55 | F, bil BC | 41,43 | i | 5837TC>AG | 1/5 | 1bil BC (57,72), 2BC (40,50), 1pan Ca (25), 1CC (70), 1 gall bladder Ca (75) |
| 56 | F,BC+MM | 39,61 | i | 7531C>T | 7/10 | 4 BC(31,33,56,-), 1 leukemia (61), 1 CC (64), 1MM (39), 1 LC (65) |

NOTE. F - female, M – male, Nr. – number, OC - ovarian cancer, BC - breast cancer (female), MBC - male breast cancer, CC - colon cancer, pan - pancreas, GC - gastric cancer, pro - prostate, bil – bilateral, LC - lung cancer, Ca - cancer, (-) no data available, (+) in the same individual

* Inclusion criteria classification:

1. probands with at least two first degree relatives with breast and ovarian cancer;
2. probands with only two first degree relatives of breast cancer where one must be diagnosed less than 50 years of age and;
3. individual patients with breast and ovarian cancer, bilateral breast cancer, breast cancer diagnosed before the age of 40 and male breast cancer without any other cancer in the family.
